# Serum 25-hydroxyvitamin D, serum calcium and vitamin D receptor (VDR) polymorphisms in a selected population with lumbar disc herniation—A case control study

**DOI:** 10.1371/journal.pone.0205841

**Published:** 2018-10-24

**Authors:** Niroshima Dedunu Withanage, Sunil Perera, Hemantha Peiris, Lohini Vijayendran Athiththan

**Affiliations:** 1 Department of Allied Health Sciences, Faculty of Medical Sciences, University of Sri Jayewardenepura, Nugogoda, Sri Lanka; 2 The Central Hospital, Colombo 8, Sri Lanka; 3 Department of Biochemistry, Faculty of Medical Sciences, University of Sri Jayewardenepura, Nugegoda, Sri Lanka; University of Crete, GREECE

## Abstract

**Background:**

Association of Vitamin D receptor (VDR) polymorphisms with lumbar disc herniation (LDH) have been identified in several ethnic groups globally. Despite abundant sunlight, vitamin D deficiency is reported in many tropical countries. As vitamin D is a key modulator for intestinal calcium absorption, low vitamin D could contribute to low serum calcium leading to abnormalities of skeletal homeostasis. Therefore, present study was aimed to study the association of serum 25-hydroxyvitamin D (25(OH)D), serum calcium and VDR polymorphisms in a selected Sri Lankan population.

**Materials & methods:**

A case control study was conducted in 119 participants (cases = 51: controls = 68). Serum 25(OH)D levels were measured using ELISA. The VDR polymorphisms (*Fok I* and *Taq I*) were detected by polymerase chain reaction followed by restriction fragment length polymorphism.

**Results:**

Findings indicated a significantly low (p = 0.000) 25(OH)D levels in cases (18.7±3.7 ng/mL) compared to controls(25.5±9.8 ng/mL) while 25(OH)D in both groups were below the reference range. Mean serum calcium levels in both groups were within normal reference range and was not significantly different among groups. Statistically significant association was not observed between VDR *Fok I* polymorphisms among cases and controls. Although *Fok I* polymorphism genotypes were in Hardy-Weinberg equilibrium (HWE), *Taq I* genotypes in controls violated HWE.

**Conclusion:**

Present study confirms that insufficient serum 25(OH)D levels in cases have major contribution to LDH. VDR *Fok I* polymorphisms did not have any significant association with LDH in Sri Lankan ethnicity.

## Introduction

Vitamin D is a steroid hormone which has variety of functions in bone, muscle and cartilage metabolism. It also facilitates calcium and phosphorous absorption from human gut and kidneys through the action of parathyroid hormone (PTH) and calcitonin [1–4]. High levels of serum vitamin D reduce PTH and stimulate bone formation by its direct action on osteoblasts. Therefore, deficiency in vitamin D is considered as a strong predictor of bone metabolism disturbances [1]. Decreased vitamin D levels were associated with several health issues such as, derangement of calcium metabolism, matrix calcification, low bone density and cartilage turnover, osteoporosis, osteomalacia, osteoarthritis, rickets, neurodegenerative diseases and chronic low back pain [5–9]. Major portion of required vitamin D requirement is synthesized by the skin through the photolysis of 7-dehydrochoelsterol upon exposure to sunlight in humans [4, 10]. However, several studies conducted in tropical countries such as, India, China, Turkey, Malaysia, Iran and Saudi Arabia revealed that there is a high prevalence of insufficient vitamin D status in these countries irrespective of unlimited abundant sun light [1, 2, 5, 8].

In human body, vitamin D is converted to 25-hydroxyvitamin D (25-(OH)D) which in turn is converted to its active metabolite 1,25-dihydroxyvitamin D (1,25-(OH)_2_D) [4]. Function of vitamin D is meditated by vitamin D receptor (VDR), a steroid nuclear receptor which is located on chromosome 12 with 5.6 kb and it was the first reported gene associated with degenerative diseases of the disc [11, 12]. The VDR helps to regulate calcium homeostasis, bone mineralization and remodeling. Serum calcium and vitamin D has many vital roles in bone metabolism and skeletal homeostasis. It is well documented that vitamin D is the prime modulator of intestinal calcium and phosphorous absorption, hence, deficiency in vitamin D leads to deficiency in serum calcium that leads to many skeletal pathologies including fractures [13].

Some studies have reported expression of VDR increases chondrocyte differentiation, proliferation and maturation of cartilage. Studies have also reported the presence of VDR in intervertebral disc (IVD) cells. Studies have indicated a prominent role of vitamin D active metabolites in disc cells promoting regulation of cell proliferation, matrix gene expression with specific cytokines and protein production. Studies have explained that several pathological conditions in IVD are associated with alterations of vitamin D homeostasis. This could be explained by the pleotrophic effect of vitamin D and its involvement in bone and disc metabolism [12]. Although, the exact mechanism of decreased VDR function in IVD is poorly understood, literature suggests that vitamin D might play a role in sulphate concentration of proteoglycans (PG) and also determine the degree of sulphation in PG, which maintains the stability of the disc [14].

However, recent epidemiologic studies on lumbar disc herniation (LDH) have highlighted that hereditary factors could contribute to the development of LDH with relatively minor contributions with environmental, occupational and behavioural factors [11, 14, 15]. Numerous genes such as VDR, aggrecan, collagen type I, IX and XI, cartilage intermediate layer protein, matrix metalloproteinases (MMPs) 1,2 and 3, interleukin1,6 and several other genes have been studied in association with lumbar disc degeneration (LDD) and LDH. However, VDR polymorphisms had been investigated extensively in many ethnic groups including Finnish, Indian, Italian, Egyptian, British, Chinese, Australian and Japanese [16–19].

Despites the geographic location of Sri Lanka in the tropical region, initial investigations of the present study detected low serum 25(OH)D levels in cases (patients with LDH) as well as in controls. This was significantly lower in the cases when compared to controls. However, findings of low vitamin D levels in Sri Lankan subjects further supports the reported high prevalence of vitamin D deficiency in other tropical countries. Therefore, above findings further strengthen that genetic predisposition attributes for low vitamin D levels and development of LDH in subjects with vitamin D deficiency.

Although, studies on VDR have focused on selected variants of VDR including *Taq I* (rs731236), *Fok I* (rs 2228570) and *Apa I* (rs 7975232) restriction sites [11, 14, 16], *Fok I* and *Taq I* polymorphisms of VDR have been studied comprehensively in relation to lumbar disc degeneration and herniation (LDHD) in different ethnic groups. However, to our knowledge, there are no reported studies investigating the association of 25(OH)D and VDR polymorphisms with regard to LDH in Sri Lankan or other South Asian populations. This is the main strength of the present study. Therefore, based on these evidences, the present study focused on the association of 25(OH)D, serum calcium and VDR polymorphisms in a selected Sri Lankan population with LDH.

## Materials and methods

### Study design and setting

The study was carried out at the Faculty of Medical Sciences, University of Sri Jayewardenepura and GENETECH Institute, Colombo 08, Sri Lanka. Cases were recruited from a hospital in the district of Colombo, while controls subjects represented several districts of Sri Lanka. The study protocol was approved by the Ethics Review Committee of Faculty of Medical Sciences, University of Sri Jayewardenepura, Sri Lanka (29/14). After explaining the study protocol, informed written consent was obtained from all individual participants.

### Study subjects

A case-control design, with a total of 119 Sri Lankan subjects age ranging between 18–74 years were enrolled in this study. Inclusion criteria for cases were the presence of lower back pain (LBP) and LDH confirmed by Magnetic Resonance Imaging (MRI), while inclusion criteria for controls were subjects without LBP at least during the past one month prior to the study and who have not undergone lumbar discectomy. The concomitant presence of other bone disorders such as osteoarthritis, osteoporosis and supplementation on vitamin D, pregnancy, malignancies were exclusion criteria for both cases and controls.

### Sample size calculation

$$n=\frac{K\left[\pi 1\left(1-\pi 1\right)+\pi 2\left(1-\pi 2\right)\right]}{\delta 2}$$

Where,

n = The sample size in one group

K = A function of chosen significance level and chosen power

The power of the study will be 80% and the 2 sided significance level will be 0.05. The K value is for the power and significance level is 7.8.

The characteristic of interest–polymorphism of VDR gene.

π1 = Proportion of subjects with the disease who are having the characteristic of interest.

π 2 = Proportion of normal subjects who are having the characteristic of interest.

δ = (π1-π2)

n = 45 (each in test and control)

### Serum 25-hydroxyvitamin D analysis

Serum 25(OH)D levels were determined by Enzyme Linked Immunosorbent assay (ELISA) using commercially available ELISA kit (DRG Instruments, USA). Absorbance was measured at 450 nm. In accordance to the kit’s instruction, serum 25(OH)D concentration of 30 ng/mL–100 ng/mL was considered as sufficient level of 25(OH)D.

### Serum calcium analysis

Serum calcium was measured using automated Konelab 20 XT clinical analyzer. Kit standard R2 (calcium 10 mg/dL: 2.5 mmol/L, Biolabo, France) was used to prepare the calibration curve.

### Genomic DNA analysis

Peripheral blood was collected and genomic DNA was extracted using Promega Wizard DNA extraction kits. The polymorphisms of VDR (*Fok I* and *Taq I*) genes were detected with polymerase chain reaction (PCR) assay (Applied Biosystem 2720 thermal cycler, Life Technologies, Singapore). Gel imaging was done by E-Gel imager (Life Technologies, Singapore). The primers used for VDR gene amplification were obtained from previous literature.

*Fok I* [20, 21]

Forward

5'-AGC TGG CCC TGG CAC TGA CTC TGC TCT -3'

Reverse

5'-ATG GAA ACA CCT TGC TTC TTC TCC CTC-3-'

*Taq I* [22]

Forward

5'-CAG AGC ATG GAC AGG GAG CAA -3'

Reverse

5'-GCA ACT CCT CAT GGC TGA GGT CTC -3'

#### *Fok I* polymorphism

The PCR cycle conditions were: Denaturation at 94 °C for 5min, followed by 35 cycles at 94 °C for 60 s, 61 °C for 30 s and 72 °C for 1 min and one final extension at 72 °C for 7 min. The PCR reaction mixture (50 μL) consisted of 100–200 ng of purified genomic DNA, 5 μL of PCR buffer (with 20 mM MgCl_2_), 3 μL of forward and reverse primers (2–6 pmol), 10 mM dNTPs (1 μL) and 1.5 units of Taq DNA polymerase. Amplified DNA was electrophoresed on 2% agarose gel to verify the size of amplicons (265 bp).

The mixture for restriction enzyme digestion was prepared by adding; 2μL of 10X buffer (50 mM Potassium acetate, 20 mM Tris acetate, 10 mM Magnesium acetate, 100 μg/mL BSA, pH 7.9), 0.4 μL of *Fok I* enzyme (New England Bio Labs, UK), 6 μL of amplified PCR product (265 bp), 0.02 μL of 0.5% Tween 20 (Promega, USA). Sterile distilled water (11.6 μL) was used to make the final volume to 20 μL. The mixture was incubated at 37 °C for 1 hour and resolved initially at 50 V for 5 minutes and then kept in 100 V for 2 hours on 2% agarose gel and stained with ethidium bromide. Restriction fragments were visualized under ultra violet (UV) light in E-Gel imager (Life Technologies, Singapore) and was photographed.

#### *Taq I* polymorphism

The PCR cycle conditions were; Denaturation at 94 °C for 3min, followed by 35 cycles at 94 °C for 60 s, 65 °C for 60 s and 72 °C for 2 min and one final extension at 72 °C for 5 min. The PCR reaction mixture (50 μL) consisted of 100–200 ng of purified genomic DNA, 5 μL of PCR buffer (with 20 mM MgCl_2_), 2 μL of forward and reverse primers (10 pmol), 10 mM dNTPs (1 μL) and 1.5 units of Taq DNA polymerase (0.5 μL). Amplified DNA was electrophoresed on 2% agarose gel to verify the size of amplicons (740 bp).

Restriction enzyme digestion mixture for *Taq I* which consisted of 2 μL of 10X buffer (50 mM Potassium acetate, 20 mM Tris acetate, 10 mM Magnesium acetate, 100 μg/mL BSA, pH 7.9), 0.5 μL of *Taq I* enzyme (New England Bio Labs, UK), 5 μL of amplified PCR product (740 bp). Sterile distilled water (11.6 μL) was used to make the final volume to 20 μL. The mixture was incubated at 65 °C for 15–20 minutes and resolved initially at 50 V for 5 minutes followed by 100 V on 2% agarose gel for 2 hours and was stained with ethidium bromide. Restriction fragments were visualized under UV light in E-Gel imager (Life Technologies, Singapore) and was photographed.

### Statistical analysis

The frequency distribution of VDR polymorphisms and the allelic frequencies in LDH subjects and controls were compared using Chi-squared tests. Hardy-Weinberg equilibrium (HWE) test for VDR polymorphisms were conducted using online HWE calculators. All statistical analyses were performed using SPSS 20.0 version. Differences among groups were evaluated using independent t-test for continuous variables and p value <0.05 was considered as statistically significant.

## Results

### Characteristics of study subjects

Results of the present study indicated that mean age for cases and controls were 41.3±14.6 and 43.3±15.4 years respectively (Table 1). Among the study population similar male and female representation was considered. Males in cases and controls were respectively 52.9% and 51.5%. Majority of subjects in both cases (92.1%) and controls (97.1%) had limited exposure to the sun light. Majority were Sinhalese in both groups and they did not use out fits that covered the entire body.

**Table 1:** Characteristics of study subjects.

Variables	Cases (n = 51)	Controls (n = 68)
Mean age (±SD) (years)	41.3±14.6	43.3±15.4
BMI (kg/m^2^)	28.2±5.7 (n = 44)	23.5±3.4(n = 59)
Exposure to sunlight		
a)Limited exposure (Engaged in in-door activities)	47 (92.1%)	66 (97.1%)
b)Abundant exposure (Engaged in out-door activities)	04 (7.8%)	02 (2.9%)
Race		
a)Sinhala	40 (78.3%)	65 (95.6%)
b)Tamil	03 (5.9%)	02 (2.9%)
c)Muslim	08 (15.7%)	01 (1.4%)

### Serum 25-hydroxyvitamin D and serum calcium levels in cases and controls

Though cases and controls had low level of serum 25(OH)D levels, 25(OH)D concentrations were significantly low (p = 0.000) among cases than controls (Table 2). Mean serum calcium level in both cases and controls were within the normal reference range (8.6–10.4 mg/dL) and no significant difference was observed between cases and controls (Table 2).

**Table 2:** Serum 25-hydroxyvitamin D and serum calcium concentrations in study subjects.

	Cases (n = 51) Mean±SD	Controls (n = 68) Mean±SD	p value
Serum 25(OH)D (ng/mL)^a^	18.7±3.7	25.5±9.8	0.000*
Serum calcium (mg/dL)^b^	9.8±1.8	10.4±1.8	0.093

^a,b^Reference range for 25(OH)D: 30–100 ng/mL (insufficiency: 10–29 ng/mL) and serum calcium: 8.6–10.4 mg/dL)

### VDR Genotypes and alleles in cases and controls

**Fig 1:**
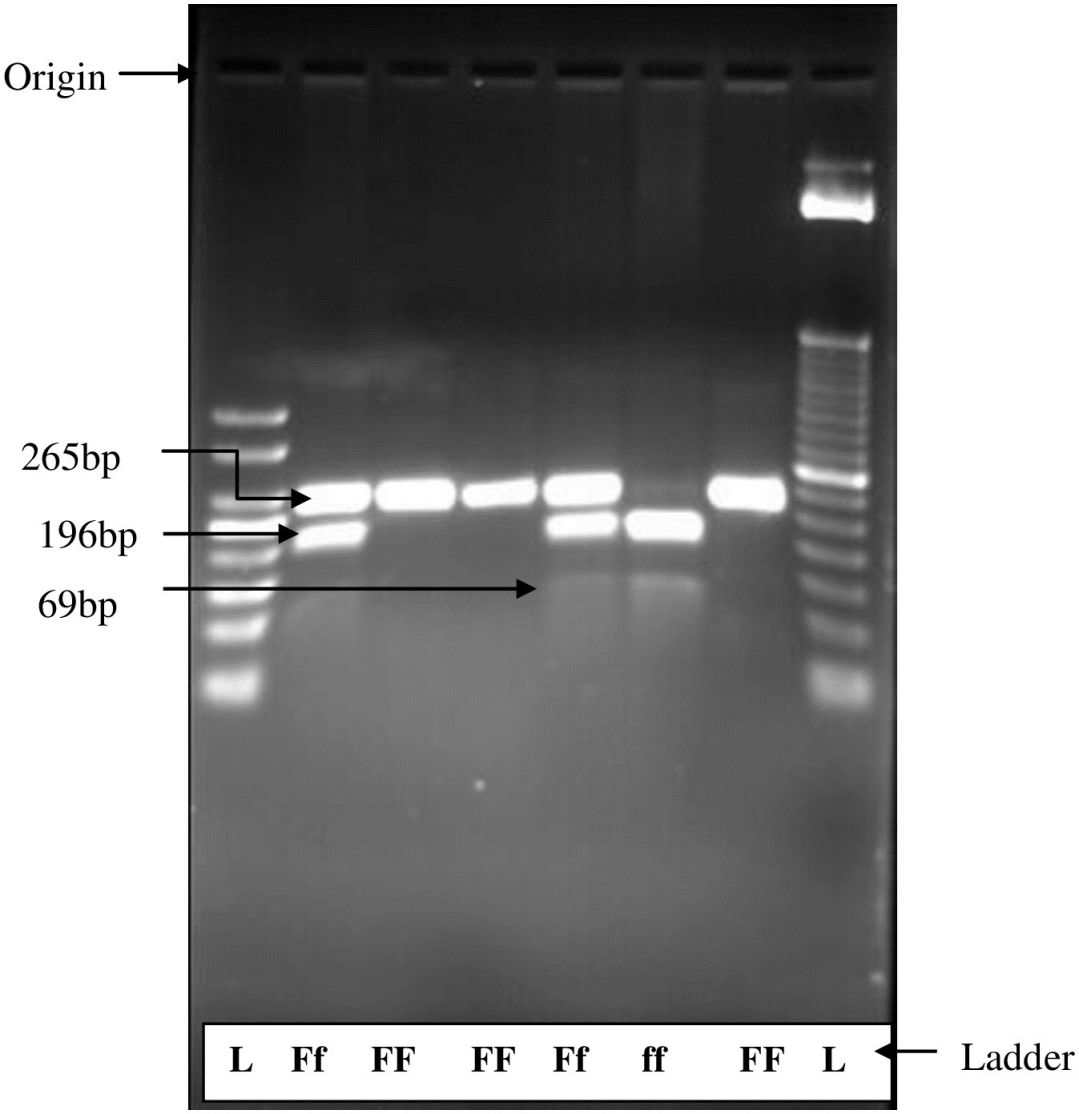
Agarose gel electrophoresis for restriction fragment length polymorphism of VDR *Fok I*.

**Fig 2:**
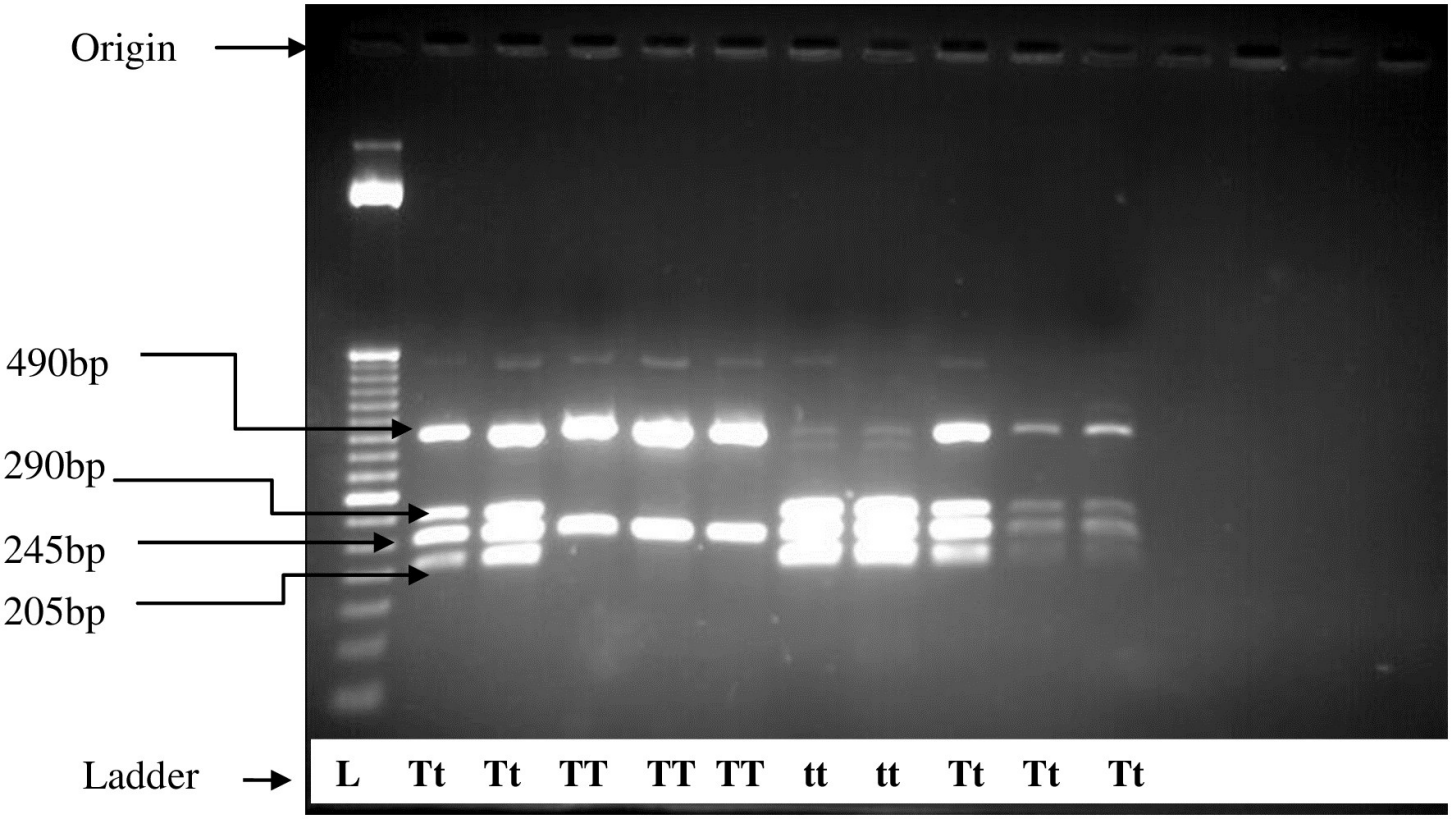
Agarose gel electrophoresis for restriction fragment length polymorphism of VDR *Taq I*.

The restriction fragment length polymorphism (RFLP) genotypes and allele frequencies are indicated in Tables 3 and 4 while Figs 1 and 2 show RFLP fragment patterns yielded for *Fok I* and *Taq I* polymorphisms respectively. The PCR amplified fragment of interest for *Fok I* yielded 69 bp, 196 bp and 265 bp length fragments on 2% agarose gel electrophoresis. Homozygous wild type FF had no recognition site and yielded uncut 265 bp band, while homozygous ff has one restriction site and yielded two bands with size of 196 bp and 69 bp. Heterozygous Ff yielded all the above 3 bands (Fig 1).

**Table 3:** Distribution of vitamin D receptor *Fok I* genotype and alleles in cases and controls.

Variable		Cases n (%)	Controls n (%)	Odds ratio (95% CI)	χ^2^	p value
VDR *Fok I* genotypes	FF	34 (66.7)	38 (55.8)	0.69 (0.32–1.49)	0.9	0.365
	Ff	16 (31.4)	26 (38.2)	0.32 (0.03–3.04)	1.05	
	ff	1(1.9)	4 (5.8)	0.22 (0.02–2.01)	2.9	
VDR *Fok I* alleles	F	84 (82.4)	102 (75)			
	f	18 (17.6)	34 (25)			

**Table 4:** Distribution of vitamin D receptor *Taq I* genotype and alleles in cases and controls.

Variable		Cases n (%)	Controls n (%)
VDR *Taq I* Genotypes	TT	31 (60.8)	25 (36.8)
	Tt	16 (31.4)	39 (57.4)
	tt	4 (7.8)	4 (5.9)
VDR *Taq I* alleles	T	78 (76.5)	89 (65.4)
	t	24 (23.5)	47 (34.6)

All the genotype frequencies analyzed for *Fok I* polymorphisms were in HWE (χ^2^ = 0.13). Among the study population homozygous wild type FF genotype was common for *Fok I* polymorphisms in both cases (66.7%) and controls (55.8%), while mutant ff genotype showed the least occurrence in both cases (1.9%) and controls (5.8%). The allelic frequencies for F allele were 82.4% and 75% for cases and controls respectively, whilst f allele represented 17.6% and 25% respectively in cases and controls. The distribution of each allele in subjects with disc herniation was compared with that of the controls. However, there was no significant difference between distributions of *Fok I* polymorphism with LDH (Table 3).

In the analysis of *Taq I* digestion, the PCR amplified fragment of interest yielded 205 bp, 245 bp, 290 bp and 490 bp in length in 2% agarose gel electrophoresis. Homozygous TT has one recognition site and gave 2 bands at 245 bp and 495 bp, while homozygous tt generated an additional restriction site giving 3 bands of 290 bp, 245 bp and 205 bpin length. Heterozygous Tt yielded 4 bands at 205 bp, 245 bp, 290 bp and 495 bp (Fig 2).

The genotypes of *Taq I* polymorphisms for cases were in accordance with HWE. However, genotypes for controls violated the HWE. Therefore, further analysis on *Taq I* polymorphisms was not done (Table 4).

## Discussion

Poor serum vitamin D levels have been proven globally. Interestingly, similar findings have been reported in tropical countries such as India, Iran, Turkey, China and Malaysia. Vitamin D has several important functions in skeletal and non-skeletal metabolism which exerts its action through VDR. Major cause for chronic low back pain is LDH. This has shown promising association with genetic influences rather than conventional risk factors [12, 23–25]. There are several reported studies on the association of VDR polymorphisms with LDHD which has been validated in several ethnic groups worldwide [12, 14, 26, 27]. An initial investigation of the present study showed a decreased serum vitamin D level in both cases and controls. Since VDR plays an important role in vitamin D metabolism, investigators of the present study investigated the VDR genetic polymorphisms with serum calcium and vitamin D with LDH. To our knowledge this is the first study to evaluate the association of VDR gene polymorphisms with LDH in Sri Lankan population.

### Association of serum 25-hydroxyvitamin D and serum calcium concentrations in study subjects

Surprisingly, insufficient mean serum 25(OH)D levels in both cases and controls were observed in the present study despite of adequate sunlight in Sri Lanka’s tropical climate. However, similar sub optimal levels of 25(OH)D have been recorded in many countries in the same region; several parts of India, Malaysia, Turkey, China, Saudi Arabia and Iran. Although the main source of production of vitamin D is regulated by the cutaneous synthesis under the influence of sunlight, there are some factors which may affect the cutaneous vitamin D production such as, skin pigmentation, aging, use of sun screen, clothing pattern, exposure to sun, latitude and season. In the present study, majority of both cases (92.1%) and controls (97.1%) were not exposed to sun light as they were engaged in in-door activities by means of employment, studying or other non-specific activities. Rapid modernization and urbanization in a developing country like Sri Lanka has further rendered people for a minimum exposure to sunlight. Therefore, it is suggested that although Sri Lanka is a tropical country, limited exposure to sun light may be a critical cause for the insufficient vitamin D in the present study. Further, out fits that cover majority of the body diminishes the amount of UV rays, which is regarded as a significant contributory factor for the production of cutaneous vitamin D production. Therefore, it is evident that in some societies or religions such as Muslims, who wear extensive coverings of clothes have widespread of vitamin D deficiencies, especially in some middle-east countries [28]. Although Sri Lanka is a multi-cultured country, in the present study there were limited number of Muslim and Tamil subjects in both cases (Muslims; n = 8 and Tamils; n = 3) and controls (Muslims; n = 1 and Tamils; n = 2). However, all the subjects participated in the study did not use any specific coverings irrespective of their religion. Therefore, clothing is not a contributory factor for the insufficient vitamin D levels in the study population [29].

A study conducted in rural female population in India has revealed that 88.6% adolescent girls and 74% of pregnant women had shown 25(OH)D deficiency [9]. Although, some studies hypothesized that rural population has excellent sun exposure [9], present study did not consider the place of residence (urban or rural), as majority of present study population had limited exposure to sun light irrespective of the place of residence. Further, studies have reported that obesity plays a role in vitamin D deficiency [30], accordingly in the present study the cases had mean BMI above the normal range, and 25.4% were obese, however, the mean BMI of the controls were within the normal range.

A multi centered study conducted in different urban areas of Iran has also found severe to moderate 25(OH)D deficiency in both men and women [1]. Though the role of 25(OH)D in LDH is not well understood, it is believed that vitamin D plays a role in sulphate concentration of PG and also determines the degree of sulphation in PG which helps to maintain the stability of the IVD [14]. Present study analysis expressed a statistical significance in the mean 25(OH)D among cases and controls (p = 0.000), where, cases had severe insufficiency in 25(OH)D levels (18.7 ng/mL) compared to controls (25.5 ng/mL) (Kit reference value: insufficiency indicates 10–29 ng/mL). Present finding is strengthened by several similar reported studies. A study conducted in patients with degenerative changes in the lumbar spine (n = 110) revealed high prevalence of vitamin D deficiency with the cohort of patients [31]. In another study similar observation to the present study was highlighted in patients with chronic lower back pain and leg pain following lumbar spinal stenosis. Above study recorded a low mean 25(OH)D of 15.9±7.1 ng/mL. Further, they have found majority of patients (74.3%) as vitamin D deficient subjects. The study also revealed that there was a significantly higher prevalence (p≤0.001) of vitamin D deficiency in patients with severe type of back and leg pain when compared to mild and moderate types of back or leg pain. Further, authors have recommended the estimation of 25(OH)D in patients with lumbar spinal stenosis that are associated with severe back or leg pain. In addition they have recommended combination of vitamin D and calcium as a treatment option in this category of patients [32].

Further, a study reported in Saudi Arabia also found similar observations to the present study, indicating that 83% of the patients with LBP suffered from 25(OH)D deficiency [28].

As vitamin D determines the degree of sulphation in PG in the disc cells, which is vital in maintaining the disc stability [16], it is of great importance to explore further to find out the reason for the reduction in vitamin D in cases, as this may attribute for the alteration in disc stability leading to LDH.

Importantly, findings of the association of vitamin D with LDH in the present study well correlated with the reported studies on the role of vitamin D in protection of IVD cells against neurotoxic agents [33]. These findings also explained the role of vitamin D in down regulation of pro-inflammatory markers such as cytokines and up regulation of anti- inflammatory cytokines in LDH and LDHD. Therefore, the insufficiency of vitamin D may trigger the secretion of cytokines leading to inflammation of the disc and also elevate inflammatory markers such as C-reactive protein and high sensitivity C-reactive protein. As indicated in reported literature the pain associated with LDH is not only due to compression of spinal nerve but also can be explained by the ability of vitamin D in down regulating the pain related cytokines.

Although, Sri Lanka being a country with adequate exposure to sunlight, findings of vitamin D in the present study provided valuable insight for the association of vitamin D in LDH. Therefore, even in a tropical climate screening for vitamin D deficiency and supplementations of vitamin D should be practiced mandatorily in patients with LDH and low vitamin D.

Although, 25(OH)D levels were insufficient in the study population, serum calcium levels in both groups were within the normal reference range with no significant difference between groups suggesting that serum calcium concentration does not play an important role in development of LDH. However, PTH and calcitonin secreted by the thyroid gland are also responsible in regulating serum calcium. Therefore, in spite of low 25(OH)D levels PTH has contributed to maintain normal serum calcium level in the present study. Although, estimation of PTH levels in study population could have added more glamour to the study, this was not carried out due to high cost of PTH laboratory investigations.

#### Prevalence of vitamin D receptor *Fok I* polymorphism in the study sample

In the present study, prevalence of wild type homozygous F allele in cases was 82.4% whereas prevalence of risk f allele was 17.6%. Controls had 75% and 25% prevalence for F and f alleles respectively. There was no significant difference of *Fok I* polymorphisms between cases and controls (p = 0.365). However, our prevalence rates are not compatible with the genotype and allelic frequencies of different reported studies among different ethnic groups. Our study indicated higher percentage for the occurrence of F allele and lower frequency for f allele when compared to other reported studies.

A similar study conducted in Italy (n = 487) has reported genotype and allele frequencies among cases; FF-43.8%, Ff-45.0%, ff-11.2%, F-66.3% and f-33.7%, whereas in controls; FF-40.5%, Ff-45%, ff-14.5%, F-63% and f-37% [12]. Similar frequency distribution was reported in Finnish twins (n = 85 pairs, FF-28%, Ff-58%, ff-14%, F-60%, f-40%), Finnish controls (n = 56, FF-44.6%, Ff-46.4%, ff-8.9%, F-67.9%, f-32.1%), Turkish healthy subjects (n = 150, F-67%, f-33%), German healthy women (n = 2596, FF-38.5%, Ff-46.3%, ff-15.2%, F-62%, f-38%), Mexican patients with lumbar disc degeneration and controls [(cases: n = 100, FF-20%, Ff-65%, ff-15%) (controls: n = 100, FF-32%, Ff-51%, ff-17%)] [12, 24].

Although, there are no reported data available in the Sri Lankan context regarding the prevalence of VDR gene polymorphisms, similar genetic polymorphisms have been studied in some countries in Asia namely China and Japan [26, 27, 34, 35]. However, there were no reported studies of VDR polymorphisms with LDH pertaining to South Asian countries which remains as the major value of the present study. Hence, data are not available for South Asian region, comparison of the present study was not done. Perhaps, variation in Sri Lankan population from the rest of the world may signify the true impact of ethnicity. Thus, this kind of study may open up the window for future establishment of epidemiological and clinical databases. Hence, similar studies are needed to be carried out in order to investigate the true prevalence in Sri Lanka as well as other South Asian countries in order to provide a definite confirmation.

#### Prevalence of vitamin D receptor *Taq I* polymorphism in the study sample

Although, the genotypes and allelic frequencies of *Taq I* for cases were in agreement with HWE, controls violated the HWE. Therefore, further analysis of *Taq I* could not be performed. Regarding cases, prevalence of T allele in cases was 76.5% whereas t allele was 23.5%. Prevalence of *Taq I* genotypes of the present study was in accordance with the prevalence of those in other reported studies with LDH and LDHD. Similarly, genotype frequencies of *Taq I* polymorphisms on cases were in accordance with similar studies carried out in patients with LDHD in Asian countries as well as other countries. According to a study conducted by Kawaguchi et al (2002) in Japanese adults with or without low back problems (n = 205), have observed TT-74%, Tt-26%, tt-0% which did not have a significant difference with LDHD. A similar observation was recorded in a case control study of patients with IVD degeneration in Mexican population (TT-69%, Tt-27%, tt-3%) [24]. However, contrast findings were reported in China by Yuan et al (2010). They have conducted a study in patients with lumbar disc degeneration (n = 178) and controls (n = 284) and the investigators found a higher prevalence of TT-90.1% and tt-9.86% among patients with disc degeneration and herniation [35].

Although, in the present study researchers recruited more than the calculated sample size (n = 45 for each study group), investigators strongly believe that findings regarding *Taq I* polymorphisms of VDR should be repeated with higher sample size in order to arrive at a proper conclusion as no reported studies on *Taq I* polymorphisms in Sri Lankan population is available in literature. Investigators of the present study also suggest that violation of HWE in *Taq I* polymorphisms could be due to small sample size and genetic analysis of a larger population may overcome that problem. However, recruitment of higher number of cases was not feasible with the present study, which remains as a major limitation of the study.

However, investigators of the present study evident that similar genetic studies have been conducted in smaller populations in Japan by Oishi et al (2003) (n = 60) and Noponen-Hietala et al (2003) (n = 85) on VDR *Taq I* polymorphism in disc degeneration. Similar to our findings authors have reported that there was no correlation with the presence of different genotypes of *Taq I* and *Fok I* with disc degeneration [34, 36]. However, these findings may be attributed to the small sample size in the above studies.

### Association of vitamin D receptor gene polymorphisms with lumbar disc herniation/degeneration

VDR mediates vitamin D, which in turn regulates bone mineralization, remodeling and calcium homeostasis. VDR polymorphisms, *Fok I* and *Taq I* also have been studied in other common bone disorders like osteoporosis and osteoarthritis in addition to LDH and LDHD.

Present study findings had no significant association between the distribution of *Fok I* genotype with cases and controls. Our findings are further strengthened by some reported studies in other countries. Similar observations were reported in a case control study conducted in Mexico. Authors have stated that there was no significant association neither with *Fok I* polymorphisms between cases and controls [OR = 0.81; (95% CI = 0.55–1.21), p = 0.183] nor with *Taq I* [OR = 0.82; (95% CI = 0.49–1.35), p = 0.262] [24].

However, several studies conducted in different populations have reported contrast findings to the present study, as the majority of studies have found significant relationship between *Taq I* and *Fok I* polymorphisms with respect to LDHD. A case control study conducted in Turkey (n = 300) in patients with LDHD indicated that there was a significant difference between tt and ff genotypes with extrusion and sequestration types of LDH (p = 0.023 and 0 = 0.008). Authors further found that genotypes TT, Tt, FF and Ff showed a significant association with protrusion type of disc herniation (p<0.001). Investigators further observed a significant association (p<0.001) between TT and FF genotypes of the VDR gene and mild forms of disc degeneration. Further, tt, ff and Ff genotypes were associated with severe forms of degeneration (p = 0.048, p = 0.004 and p = 0.041) respectively [14]. However, in the present study further classification of patients according to the type of herniation was not done. Similar observations were reported by a study conducted in Japan (n = 205). Authors of the study have commented that there was a significant difference (p = 0.037) in the distribution of *Taq I* polymorphisms with the number of levels of disc degeneration. They have further concluded that Tt genotype of VDR gene was more frequently associated with multi-level disc degeneration and herniation rather than TT genotype [26].

Videman and co-workers (1998) affirmed that there was no association with *Taq I* and *Fok I* genotypes with disc herniation (n = 170), but they further commented that MRI signal intensities of thoracic and lumbar discs with *Taq I* tt and Tt genotypes was worsen by 12.9% and 4.5% respectively than that of TT genotypes. Authors further argued that signal intensities were 9.3% and 4.3% lower in patients with *Fok I* ff and Ff genotypes, when compared to FF genotype [37].

A study conducted in Australian males (n = 282) had stated that presence of tt of *Taq I*, showed an increased risk of developing osteophytes and disc narrowing compared to normal genotype TT [38]. A case control study conducted in Italy, has found a strong association for the presence *Fok I* F allele with disc herniation [19]. Chenug et al (2006) carried out a similar study in a large Chinese population (n = 804) and discovered that t allele of *Taq I* is associated with high risk of lumbar disc degeneration [OR = 2.61 (95% CI = 1.15–5.90), p = 0.041] and occurrence of disc bulge, especially in younger individuals of less than 40 years of age [27].

Results of a study conducted as a cohort in Italian subjects (n = 487) who were confirmed for the presence of lumbar spine pathologies with MRI has revealed that presence of FF genotype and F allele of *Fok I* of VDR represented a 2 fold risk of developing disc herniation, whereas f allele of *Fok I* has been recognized as protective [12].

Although, we could not find a significant association between the genotype of *Fok I* with the degree of degeneration, Eser et al (2010) found a significant association (p <0.001) for mild disc degeneration with FF and TT genotypes. They also discovered a significant similar observations with severe disc degeneration in tt, Ff and ff genotypes (p = 0.048, p = 0.004 and p = 0.041) [14].

However, present study could not establish an association of VDR gene polymorphisms (*Taq I* and *Fok I*) with LDH. Perhaps this may be due to true genetic variations in Sri Lankan population or may be due to limited sample size. In addition the comparison of data in available literature remains challenging as the study designs and ethnic differences in various research studies are different. In general, genetic risk factors might interact with behavioural and environmental factors, thus enhancing LDH. More importantly findings of the present study would provide beneficial foundation for future studies as there are no literature available for VDR polymorphism, not only in relation to LDH in other South Asian countries but also for other diseases in Sri Lanka as well. Hence, the present study adds to the literature as the first study on association of VDR genetic polymorphisms with LDH in South Asian region. As genetic variations may vary from one ethnicity to another, authors of the present study have a strong feeling that these findings regarding VDR polymorphism and LDH exhibit the true association and prevalence of its own.

## Conclusion

Present study showed that severe insufficient serum 25(OH)D level in cases has a significant contribution to LDH. Present study proves that there is no significant association of VDR *Fok I* polymorphism with LDH in Sri Lankan ethnicity. Therefore, even in a tropical climate screening for vitamin D defeciency and treatment with supplements should be practiced as mandatory in patients with LDH.

## Supporting information

S1 Table Descriptive statistics and independent T test of study subjects. (DOCX)

S2 Table Classification of race and exposure to sunlight among study subjects. (DOCX)

S3 Table Distribution of vitamin D receptor polymorphisms (*Fok I* and *Taq I*) genotypes among study subjects. (DOCX)
