# Supplementary material for: Serum 25-hydroxyvitamin D, serum calcium and vitamin D receptor (VDR) polymorphisms in a selected population with lumbar disc herniation—A case control study
Source: PLoS One. 2018 Oct 24;13(10):e0205841. doi: 10.1371/journal.pone.0205841 (PMC6200232; doi:10.1371/journal.pone.0205841)
Supplement: S1 Table — (DOCX) [file pone.0205841.s001.docx]

**S1 A Table Descriptive statistics of cases**

| **Descriptive Statistics** | | | | | |
| --- | --- | --- | --- | --- | --- |
|  | N | Minimum | Maximum | Mean | Std. Deviation |
| BMI | 44 | 19.63 | 48.71 | 28.1889 | 5.75337 |
| calcium | 51 | 7.20 | 18.30 | 9.8392 | 1.79545 |
| vitamin D | 51 | 8.1 | 27.0 | 18.716 | 3.6572 |
| Age (yrs) | 51 | 18 | 70 | 41.33 | 14.634 |
| Valid N (listwise) | 44 |  |  |  |  |

**S1 B Table Descriptive statistics of controls**

| **Descriptive Statistics** | | | | | |
| --- | --- | --- | --- | --- | --- |
|  | N | Minimum | Maximum | Mean | Std. Deviation |
| BMI | 59 | 15.06 | 34.26 | 23.5108 | 3.45929 |
| calcium | 68 | 8.30 | 19.80 | 10.4059 | 1.81378 |
| vitamin D | 68 | 13.5 | 95.5 | 25.475 | 9.8048 |
| Age (yrs) | 68 | 21 | 79 | 43.29 | 15.407 |
| Valid N (listwise) | 59 |  |  |  |  |

**S1 C Table Independent sample T test for cases and controls**

| **Independent Samples Test** | | | | | | | | | | |
| --- | --- | --- | --- | --- | --- | --- | --- | --- | --- | --- |
|  | | Levene's Test for Equality of Variances | | t-test for Equality of Means | | | | | | |
|  |  | F | Sig. | t | df | Sig. (2-tailed) | Mean Difference | Std. Error Difference | 95% Confidence Interval of the Difference | |
|  |  |  |  |  |  |  |  |  | Lower | Upper |
| calcium | Equal variances assumed | .018 | .893 | -1.694 | 117 | .093 | -.56667 | .33454 | -1.22920 | .09587 |
|  | Equal variances not assumed |  |  | -1.696 | 108.428 | .093 | -.56667 | .33405 | -1.22878 | .09544 |
| vitamin D | Equal variances assumed | 2.494 | .117 | -4.681 | 117 | .000 | -6.7593 | 1.4440 | -9.6191 | -3.8995 |
|  | Equal variances not assumed |  |  | -5.221 | 90.013 | .000 | -6.7593 | 1.2946 | -9.3313 | -4.1874 |
