# Supplementary material for: Serum 25-hydroxyvitamin D, serum calcium and vitamin D receptor (VDR) polymorphisms in a selected population with lumbar disc herniation—A case control study
Source: PLoS One. 2018 Oct 24;13(10):e0205841. doi: 10.1371/journal.pone.0205841 (PMC6200232; doi:10.1371/journal.pone.0205841)
Supplement: S2 Table — (DOCX) [file pone.0205841.s002.docx]

**S2 A Table Classification of race among study subjects**

| **Race * category Crosstabulation** | | | | | |
| --- | --- | --- | --- | --- | --- |
|  | | | category | | Total |
|  |  |  | test | control |  |
| Race | sinhala | Count | 40 | 65 | 105 |
|  |  | % within category | 78.4% | 95.6% | 88.2% |
|  | tamil | Count | 3 | 2 | 5 |
|  |  | % within category | 5.9% | 2.9% | 4.2% |
|  | muslim | Count | 8 | 1 | 9 |
|  |  | % within category | 15.7% | 1.5% | 7.6% |
| Total | | Count | 51 | 68 | 119 |
|  |  | % within category | 100.0% | 100.0% | 100.0% |

**S2 B Table Exposure to sunlight among study subjects**

| **Exposure sun * category Crosstabulation** | | | | | |
| --- | --- | --- | --- | --- | --- |
|  | | | category | | Total |
|  |  |  | test | control |  |
| Exposure sun | indoor | Count | 47 | 66 | 113 |
|  |  | % within category | 92.2% | 97.1% | 95.0% |
|  | out door | Count | 4 | 2 | 6 |
|  |  | % within category | 7.8% | 2.9% | 5.0% |
| Total | | Count | 51 | 68 | 119 |
|  |  | % within category | 100.0% | 100.0% | 100.0% |
