# Supplementary material for: Serum 25-hydroxyvitamin D, serum calcium and vitamin D receptor (VDR) polymorphisms in a selected population with lumbar disc herniation—A case control study
Source: PLoS One. 2018 Oct 24;13(10):e0205841. doi: 10.1371/journal.pone.0205841 (PMC6200232; doi:10.1371/journal.pone.0205841)
Supplement: S3 Table — (DOCX) [file pone.0205841.s003.docx]

**S3 A Table Distribution of vitamin D receptor *Fok I* genotypes among study subjects**

| **Fok * category Crosstabulation** | | | | | |
| --- | --- | --- | --- | --- | --- |
|  | | | category | | Total |
|  |  |  | test | control |  |
| Fok | FF | Count | 34 | 38 | 72 |
|  |  | % within category | 66.7% | 55.9% | 60.5% |
|  | Ff | Count | 16 | 26 | 42 |
|  |  | % within category | 31.4% | 38.2% | 35.3% |
|  | ff | Count | 1 | 4 | 5 |
|  |  | % within category | 2.0% | 5.9% | 4.2% |
| Total | | Count | 51 | 68 | 119 |
|  |  | % within category | 100.0% | 100.0% | 100.0% |

**S3 B Table Distribution of vitamin D receptor *Taq I* genotypes among study subjects**

| **Taq * category Crosstabulation** | | | | | |
| --- | --- | --- | --- | --- | --- |
|  | | | category | | Total |
|  |  |  | test | control |  |
| Taq | TT | Count | 31 | 25 | 56 |
|  |  | % within category | 60.8% | 36.8% | 47.1% |
|  | Tt | Count | 16 | 39 | 55 |
|  |  | % within category | 31.4% | 57.4% | 46.2% |
|  | tt | Count | 4 | 4 | 8 |
|  |  | % within category | 7.8% | 5.9% | 6.7% |
| Total | | Count | 51 | 68 | 119 |
|  |  | % within category | 100.0% | 100.0% | 100.0% |
